# NV-plasmonics: modifying optical emission of an NV^−^ center via plasmonic metal nanoparticles

**DOI:** 10.1515/nanoph-2022-0429

**Published:** 2022-10-19

**Authors:** Harini Hapuarachchi, Francesco Campaioli, Jared H. Cole

**Affiliations:** ARC Center of Excellence in Exciton Science and Chemical and Quantum Physics, School of Science, RMIT University, Melbourne, 3001, Australia

**Keywords:** exciton-plasmon interaction, NV^−^ center in diamond, plasmonic metal nanoparticles

## Abstract

The nitrogen-vacancy (NV) center in diamond is very sensitive to magnetic and electric fields, strain, and temperature. In addition, it is possible to optically interrogate individual defects, making it an ideal quantum-limited sensor with nanoscale resolution. A key limitation for the application of NV sensing is the optical brightness and collection efficiency of these defects. Plasmonic resonances of metal nanoparticles have been used in a variety of applications to increase the brightness and efficiency of quantum emitters, and therefore are a promising tool to improve NV sensing. However, the interaction between NV centers and plasmonic structures is largely unexplored. In particular, the back-action between NV and plasmonic nanoparticles is nonlinear and depends on optical wavelength, nanoparticle position, and metal type. Here we present the general theory of NV-plasmonic nanoparticle interactions. We detail how the interplay between NV response, including optical and vibrational signatures, and the plasmonic response of the metal nanoparticle results in modifications to the emission spectra. Our model is able to explain quantitatively the existing experimental measurements of NV centers near metal nanoparticles. In addition, it provides a pathway to developing new plasmonic structures to improve readout efficiencies in a range of applications for the NV center. This will enable higher precision sensors, with greater bandwidth as well as new readout modalities for quantum computing and communication.

## 1 Introduction

Diamond is a unique material with a large optical bandgap, high stability, and biocompatibility, providing an attractive platform for many quantum technologies [1]. The negatively charged nitrogen-vacancy (NV^−^) center in diamond [2] (referred to as the *NV center* hereafter) is one of the most versatile solid-state quantum emitters known to date [2, 3]. Diamond quantum technologies based on NV centers are rapidly evolving in areas such as quantum information processing [4–7], bio-sensing [1, 8, 9], magnetometry [10–12], electrometry [13], thermometry [14], piezometry [15], and lasing [16, 17], operating even at room temperature. NV centers coupled to optical microcavities have recently gained attention as versatile building blocks for applications in quantum information processing and sensing [1, 18].

Due to the presence of strong excitation modes known as localized surface plasmons, plasmonic metal nanoparticles (MNPs) exhibit nanocavity-like optical concentration capabilities, overcoming the half-wavelength size limitation of the conventional microcavities [19–21]. It is well known that MNPs in the vicinity of a quantum emitter can modify the emission behavior via changes to the local electric field and the local electromagnetic environment of the emitter [3, 22], [23], [24], [25], [26], [27], [28]. Due to their improved light controlling prowess compared to the individual constituents [29, 30], nanohybrids comprising MNPs and emitters such as quantum dots have emerged as powerful candidates for a myriad of applications including biosensing [27], solar energy harvesting [31], quantum information processing [32], and plasmonic lasing [20, 33].

To the best of our knowledge, the first experimental demonstration of controlled coupling between a diamond nanocrystal containing a single NV center and a metal nanosphere was done by Schietinger et al. through controlled manipulation of the particles with an atomic force microscope [3]. Their experiment demonstrated that hybrid systems comprising metal nanoparticles and NV centers are robust building blocks for novel nanophotonic light sources capable of maintaining the single photon character of the emission, while remaining stable even at room temperature [3]. As almost all major potential applications of the NV center rely in some way on its optical interrogation [34], the work of Schietinger et al. provides ample motivation to understand the ability to optically control NV centers using metal nanoparticles further. Despite such promising prospects of improving and controlling NV center-based nanodevices using metal nanoparticles, the wide array of existing and potential applications based on NV centers, and more than 50 years of NV research [2], the optical properties of NV-MNP nanohybrids are still not theoretically well understood.

We present a general theoretical model for the optical interaction of the NV center with a plasmonic nanoparticle. The novelty of our model entails; extending the existing isolated NV optical model to account for the vibronic contributions of the upper (^3^ *E*) excited state, accounting for both the local electric field and decay rate modifications induced by a proximal MNP, and solving the resulting nonlinear open quantum system using a newly introduced piecewise superoperator procedure. Our model successfully explains existing optical measurements both in the presence [3] and absence [18] of a plasmonic metal nanoparticle. We reveal new insights on the tunability of NV emission spectra using metal nanoparticles, unveiling exciting avenues for future experimental investigations and for the design and control of hybrid NV-plasmonic nanodevices.

## 2 Summary of the formalism

**Figure 1:**
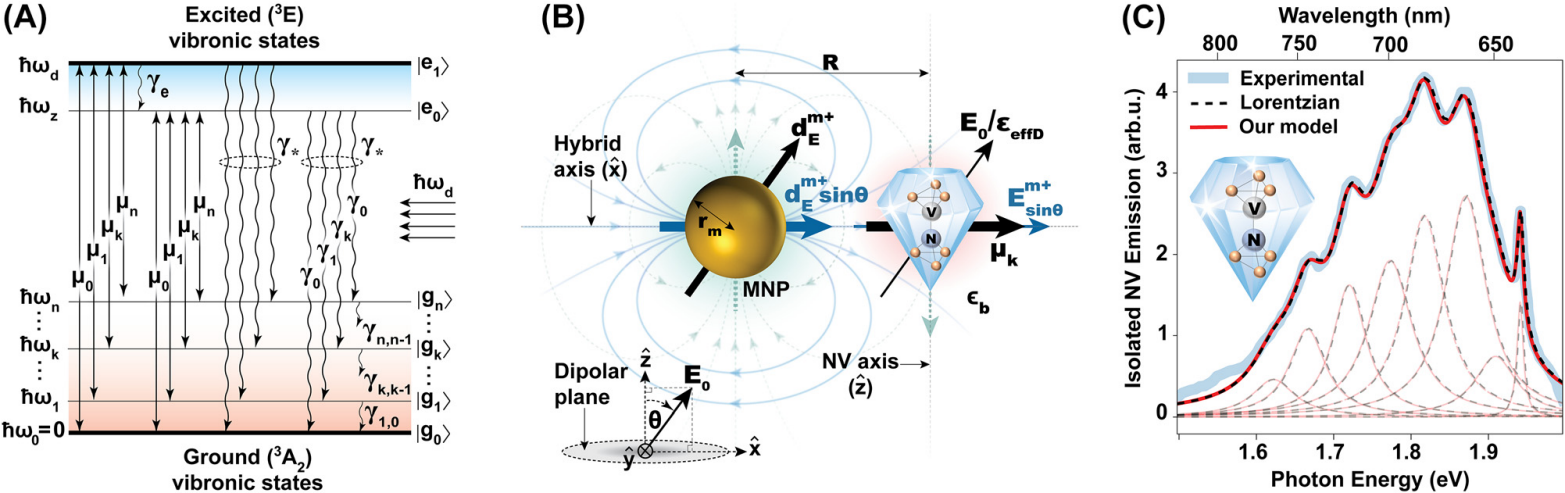
Optical model of the NV centre, example NV-MNP schematic and isolated NV emission spectra (theoretical and experimental). (A) Optical model of the NV center with *n* + 1 ground states {|*g*_k_⟩} with energies {*ℏω*_*k*_} (*k* ∈ {0, …, *n*}) and two excited states |*e*_0_⟩ and |*e*_1_⟩. The state |*e*_1_⟩ is a phenomenologically defined upper excited level resonant with the angular frequency of incoming radiation *ω*_d_. Other parameters are, spontaneous photon emission rate(s) *γ*_*k*_, dephasing rate γ_*_, ground state phonon decay rate(s) *γ*_*k*,*k*−1_, effective excited state phonon decay rate *γ*_e_, and zero phonon line (ZPL) energy *ℏω*_z_. (B) An example dimer setup where the NV-MNP hybrid axis lies along the NV dipolar plane. Optical illumination is polarized along a plane perpendicular to the NV dipolar plane. ***E***_0_ and $d_{\text{E}}^{\text{m}+}$ denote the positive frequency amplitudes (coefficients of ${\text{e}}^{-\text{i}\omega_{\text{d}}t}$ ) of the external field and the MNP dipole formed by the external field, respectively. *E*_0_ sin *θ*/*ϵ*_effD_ and $E_{\text{sin}\theta }^{\text{m}+}$ denote the screened projections of the external field and the MNP dipole response field onto the NV dipolar plane, at the NV location. (C) The experimentally measured emission spectrum of an isolated NV center in air at room temperature and the fitted Lorentzian sum reported by Albrecht et al. in [18] are denoted by the solid blue and dashed black lines. The output of our extended NV model in (A) implemented as an open quantum system is plotted in red. Individual emission lines are shown with reduced opacity. Both theoretical and experimental spectra including the fitted Lorentzians and the individual emission bands are normalized by the area of the respective total emission curve. The inset conceptually illustrates the NV center embedded in a nanodiamond.

In this work, we first combine the insights from Albrecht et al. [18] and Su et al. [35], to propose an optical abstraction for the NV center, as schematically depicted in Figure 1(A). We model the NV center as a multi-level atom with *n* + 1 vibronic levels {|*g*_k_⟩}, *k* ∈ {0, …, *n*}, in the optical ground state ^3^ *A*_2_. The number of vibronic transitions from |*g*_*k*_⟩ to the zero phonon level |*g*_0_⟩ is denoted by *k*. The lowest energy level in the optical excited state ^3^ *E* is denoted by |*e*_0_⟩. Excited state vibronic levels above |*e*_0_⟩ are represented by an effective upper excited level |*e*_1_⟩ resonant with the energy of incoming radiation. This allows capturing the ability of an NV center to be optically excited by a photon possessing higher energy than its zero phonon transition |*e*_0_⟩ → |*g*_0_⟩, for example, by a green photon. Such excitation of the NV center into |*e*_1_⟩ results in rapid nonradiative decay into the excited band edge |*e*_0_⟩, followed by a radiative transition into one of the ground levels |*g*_*k*_⟩ emitting a lower energy (red) photon in the *k*th band of the NV emission spectrum. When continuously driven with an optical field, coherent dipolar transitions |*e*_*i*_⟩ ↔ |*g*_*k*_⟩, *i* ∈ {0, 1}, arise in addition to the incoherent decay and dephasing mechanisms within and between the optical states. To accurately model the emission characteristics of the NV center, it is essential to account for both radiative and nonradiative losses conceptually depicted in Figure 1(A) via the respective Lindblad operators. In essence, we solve the abstract NV model in Figure 1(A) as an open quantum system using a Lindblad master equation-based approach, further details of which are provided in the supplementary material.

It has been reported that NV optical transitions are allowed for two degenerate dipoles orthogonal to each other, lying in the plane perpendicular to the NV (symmetry) axis [18, 34, 36]. Several theoretical and experimental studies report the ability of these dipoles to couple equally well to optical fields [37, 38] resulting in annular patterns of absorption and emission [34, 39] when illuminated with optical fields polarized in the plane perpendicular to the NV axis. This plane will be called the *dipolar plane*, hereafter. Optical electric field polarizations that do not lie along the dipolar plane have been reported to result in polarization angle-dependent anisotropy of the emission intensity [34, 38], and the observed fluorescence is expected to vanish for field polarizations exactly perpendicular to the dipolar plane, for ideal configurations [34]. In our model, each transition dipole element ***μ***_*k*_, *k* ∈ {0, …, *n*}, in Figure 1(A) is assumed to be aligned along the same effective direction defined by the aforementioned degenerate optical dipolar transitions, residing in the dipolar plane.

Optically driven ^3^ *A*_2_ ↔ ^3^ *E* transitions are strongly spin preserving [15, 40], allowing us to ignore the magnetic sublevels and non spin conserving transitions [2, 15, 40] as we focus on the NV emission spectra. Generalization of our model to include these effects (for example, in the presence of microwave magnetic fields) is conceptually straightforward, although computationally demanding.

We now consider an NV-MNP dimer illuminated by an external optical electric field with magnitude $E=E_{0}{\text{e}}^{-\text{i}\omega_{\text{d}}t}+\text{c.c.}$ , where *E*_0_ is the positive frequency amplitude, *ω*_d_ is the input frequency, *t* is time, and c.c. denotes the complex conjugate of the preceding expression. The radius of the MNP is *r*_m_ and it resides at a center separation *R* from the NV center. We obtain the laboratory (static) reference frame NV Hamiltonian below, following the NV optical model in Figure 1(A) assuming dipole–dipole type interaction between the MNP and the NV center:

(1) $$\begin{array}{ll} {\hat{H}}_{\text{NV}} & =\left(\overset{n}{\underset{k=0}{\sum }}\hbar \omega_{k}|g_{k}〉〈g_{k}|\right)+\hbar \omega_{\text{z}}|e_{0}〉〈e_{0}|+\hbar \omega_{\text{d}}|e_{1}〉〈e_{1}|\\ & \;-\overset{n}{\underset{k=0}{\sum }}\overset{1}{\underset{j=0}{\sum }}\left(\left|g_{k}〉〈e_{j}|+|e_{j}〉〈g_{k}\right|\right)\mu_{k}E_{\text{tot}}. \end{array}$$

*E*_tot_ is the projection of the total effective electric field experienced by the NV center on the dipolar plane. In the absence of an MNP, *E*_tot_ is obtainable as the projection of the input field on the NV dipolar plane, screened by the emitter material, diamond. The positive frequency amplitude (coefficient of ${\text{e}}^{-\text{i}\omega_{\text{d}}t}$ ) of *E*_tot_ in this case would be ${\tilde{E}}_{\text{tot}}^{+}=E_{0}\;\text{sin}\theta /\epsilon_{\text{effD}}$ , when *E*_0_ forms a polar angle *θ* with respect to the NV dipolar plane. The screening factor is given by $\epsilon_{\text{effD}}=\left(2\epsilon_{\text{b}}+\epsilon_{\text{D}}\right)/\left(3\epsilon_{\text{b}}\right)$ , where *ϵ*_D_ and *ϵ*_b_ are the relative permittivities of diamond and the background medium, respectively.

For the two special cases where the external field is polarized along the NV dipolar plane either parallel or perpendicular to the hybrid (NV-MNP) axis, we obtain *E*_tot_ as follows,

(2) $$\begin{array}{cc} & E_{\text{tot}}=E_{\text{tot}}^{\left(1\right)+}+E_{\text{tot}}^{\left(2\right)+}+E_{\text{tot}}^{\left(3\right)+}+\text{c.c.},\;\text{where,}\\ & E_{\text{tot}}^{\left(1\right)+}=\frac{E_{0}{\text{e}}^{-\text{i}\omega_{\text{d}}t}}{\epsilon_{\text{eff}D}},\;E_{\text{tot}}^{\left(2\right)+}=\frac{s_{\alpha }\alpha \left(\omega_{\text{d}}\right)E_{0}{\text{e}}^{-\text{i}\omega_{\text{d}}t}}{\epsilon_{\text{eff}D}R^{3}},\;\text{and}\\ & E_{\text{tot}}^{\left(3\right)+}=\frac{s_{\alpha }^{2}\alpha \left(\omega_{\text{d}}\right){\text{e}}^{-\text{i}\omega_{\text{d}}t}}{\left(4\pi \epsilon_{0}\epsilon_{\text{b}}\right)\epsilon_{\text{eff}D}^{2}R^{6}}\overset{1}{\underset{j=0}{\sum }}\overset{n}{\underset{k=0}{\sum }}\left(\mu_{k}{\tilde{\rho }}_{e_{j}g_{k}}\right). \end{array}$$

The orientation parameter *s*_*α*_ takes values 2 or −1 for the cases where the NV dipole orientation is perpendicular (NV^⊥^MNP) or parallel (NV^‖^MNP) to the MNP surface, respectively. As the plasmonic dipole of a spherical (isotropic) MNP forms along the direction of the effective field incident on it, both MNP and NV dipoles would fall along the hybrid axis in the case where *s*_*α*_ = 2. Similarly, both dipole types would be perpendicular to the hybrid axis in the case where *s*_*α*_ = −1. The slowly varying amplitude (or the rotating frame equivalent) of the off-diagonal NV density matrix element between the *j*th excited state and the *k*th ground state is denoted by ${\tilde{\rho }}_{e_{j}g_{k}}$ and *ϵ*_0_ is the permittivity of free-space. Polarizability of the MNP (in m^3^ units) at *ω*_d_ is given by *α*(*ω*_d_). For large MNPs, *α*(*ω*_d_) is modelled accounting for the finite size effects [22, 41]. The recently developed generalized nonlocal optical response (GNOR) theory [42] which accounts for the nonlocal effects is used to obtain *α*(*ω*_d_) of small MNPs. Further details of the MNP models used are outlined in the supplementary material.

From Eqs. (1) and (2), it is observable that density matrix elements enter the NV Hamiltonian through the self-feedback field component $E_{\text{tot}}^{\left(3\right)+}$ via the MNP, suggesting nonlinear evolution of the NV center (conceptually similar to the case of a quantum dot in the presence of MNPs [26, 27, 43], [44], [45]).

Using a computationally efficient piecewise superoperator based procedure; we solve the nonlinear evolution of the NV density matrix in a rotating reference frame for the special cases captured by (2), and obtain the respective NV emission spectra. In this process, we consider all decoherence mechanisms captured in Figure 1(A), as well as both electric field and emission rate modifications caused by the MNP. The derivations, parameters, and the procedures of solution and generation of emission spectra are elaborated in the supplementary material.

Our simulations reveal that, for the entire parameter region explored in this work, the contribution of the positive frequency self-feedback field component of the NV center $\left(E_{\text{tot}}^{\left(3\right)+}\right)$ is at least five orders of magnitude smaller than the screened sum of the external field and the direct dipole response field of the MNP $\left(E_{\text{tot}}^{\left(1\right)+}+E_{\text{tot}}^{\left(2\right)+}\right)$ . In such regions, we can closely approximate *E*_tot_ for any polarization orientation by projecting the screened sum of the external field and the direct dipole response field of the MNP onto the NV dipolar plane, as schematically depicted for a planar example in Figure 1(B). The required positive frequency component of the MNP direct dipole response field experienced by the NV center is obtainable under quasistatic dipole approximation as [46], $E^{\text{m}+}\approx \left[\left(3d_{E}^{\text{m}+}\cdot \hat{r}\right)\hat{r}-d_{E}^{\text{m}+}\right]/\left(4\pi \epsilon_{0}\epsilon_{\text{b}}\epsilon_{\text{eff}D}R^{3}\right)$ , where $\hat{r}$ is the unit vector of NV position relative to the center of MNP, and $d_{E}^{\text{m}+}=\left(4\pi \epsilon_{0}\epsilon_{\text{b}}\right)\alpha \left(\omega_{\text{d}}\right)E_{0}{\text{e}}^{-\text{i}\omega_{\text{d}}t}$ [19].

## 3 Results and discussion

### 3.1 Comparison with experimental observations

We first validate our extended NV center model in Figure 1(A) by comparing the area normalized emission spectrum generated for an isolated NV center in air against the experimental measurements and Lorentzian fits by Albrecht et al. [18]. All three versions of spectra closely overlap as observable in Figure 1(C). Throughout this work, we use the set of NV parameters reported by Albrecht et al. [18] for a single NV center in a nanodiamond at room temperature.

We then abstractly replicate the NV-MNP dimer setup of a single NV center coupled to an MNP by Schietinger et al. [3], and compare their experimental observations to the output of our model. In their dimer-based experiment, a nanodiamond (ND) hosting a single NV center is kept in close proximity to a 30 nm radius gold nanoparticle (AuNP) on the planar platform of an inverted confocal microscope. The assembled dimer is illuminated with a 532 nm input laser beam propagating perpendicular to the plane of the confocal microscope platform. Therefore, the electric field oscillations experienced by the dimer occur parallel to the aforementioned plane. The polarization of the beam is controlled (rotated) using a *λ*/2 waveplate. The exact position of the NV center inside the ND is unknown in their experiment and it could vary from 0 to ≈40 nm from the AuNP surface. They experimentally seek the *optimal configuration* (∼NV^⊥^MNP arrangement with the shortest achievable NV-AuNP separation) with the aid of atomic force microscope (AFM) based manipulations. This requires the dipolar plane of the NV center to be perpendicular to the aforementioned confocal microscope platform plane. The top-view of our abstract version of this setup is schematically depicted in Figure 1(B), where the externally incident field is polarized along the NV-MNP hybrid axis (*θ* ≈ *π*/2 radians) for the aforementioned optimal configuration in [3].

**Figure 2:**
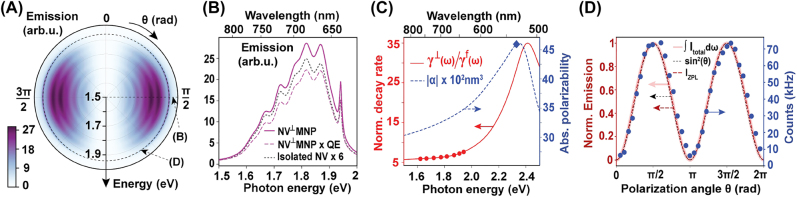
Experimental comparison of NV spectra under plasmonic influence. (A) Polar contour plot of NV emission intensities for the setup in Figure 1(B), in the presence of a gold nanoparticle of radius *r*_m_ = 30 nm located at *R* = 38 nm from the NV center in air. (B) NV Emission intensity spectrum of the same NV-MNP setup obtained at *θ* = *π*/2 radians. The solid purple line shows the total NV emission and the long-dashed purple line shows the approximate far-field NV emission accounting for the MNP induced nonradiative losses of the emitted photons. The latter is obtained by scaling the total NV emission by the quantum efficiency (QE) ∼0.78 reported for the setup in [3], which assumes a QE ∼0.99 for the bare NV center. Dotted curve depicts the spectrum of the isolated NV center magnified six times. (C) Modified decay rate spectrum (red curve) for a generic emitter oriented perpendicular to the aforementioned MNP surface, normalized by the respective free-space emission rate *γ* ^*f*^(*ω*). Red circles capture the normalized decay rates for NV^⊥^MNP (*θ* = *π*/2) case for the NV emission band peaks. The dashed blue curve depicts the absolute polarizability of the MNP, and its value at the input laser frequency (532 nm wavelength) is marked with the blue diamond. (D) Comparison of the calculated ZPL and integrated emission intensities against the experimentally detected photon counts in [3]. All emission values are normalized by the area of the isolated NV center spectrum at *θ* = *π*/2.

We present emission energy (*ℏω*) and excitation polarization angle (*θ*) sweeps of the NV emission intensity of the setup in Figure 1(B), as a polar surface plot depicted in Figure 2(A). It is observable that all emission sidebands and the zero-phonon line (ZPL) of the NV center exhibit anisotropy as a function of the polarization angle *θ* in the confocal microscope platform plane (perpendicular to the NV dipolar plane). The highest intensity of each emission peak is observed for the *θ* = 90° (NV^⊥^MNP) case. Schietinger et al. reported ∼6 times enhancement in NV^−^ emission intensity for the experimentally achieved optimal configuration, compared to the isolated NV^−^ emission and estimated a quantum efficiency of ∼0.78 for the dimer [3]. We obtain a very similar emission enhancement using our model at *R* = 38 nm. In Figure 2(B), we have shown the theoretically obtained total near-field emission intensity of the NV^−^ center in the presence of the AuNP, for the NV^⊥^MNP configuration at this center separation. The far-field emission spectrum (which can be estimated by scaling the near-field spectrum in Figure 2(B) by the quantum efficiency of ∼0.78 reported in [3]) also exhibits ∼6 times ZPL intensity enhancement compared to the isolated NV center.

At the same NV-MNP separation *R* = 38 nm, we obtain a total decay rate enhancement (the sum of modified decay rates for all transitions normalized by the sum of all isolated NV transition rates) ≈6.5 , and a ZPL decay rate enhancement ≈7.6 as observable in Figure 2(C), using the decay rate modification procedure in [22] summarized in the supplementary material. These theoretical predictions are in good agreement with the experimentally observed excited state decay rate enhancement ∼7.5 of the aforementioned optimal configuration in [3].

We then compare the dependence of NV emission intensity on the polarization angle *θ* predicted by our model against the experimentally reported variation in [3], in Figure 2(D). The theoretically predicted polarization angle dependence of emission intensity is in good agreement with the experimentally observed variation. Both theoretical and experimental emission patterns exhibit an angle dependence closely proportional to sin^2^ *θ*. The imperfect diminishing of the experimentally detected emission intensity for excitation directions along the NV axis (*θ* = 0, *π* radians) could be attributable to reasons such as imperfect alignment of polarization, sensitivity of linear polarization rotations to background effects, and particle drift [34].

The dipole approximation we use here does not account for effects such as the spatial retardation of incoming radiation, multipolar effects, and any substrate effects. However it is noteworthy that our model effectively captures the essential physics of NV-MNP interaction even with such approximations, in the parameter regime of interest. It is also important to note that we have not fitted the results of Schietinger et al. [3], but rather used the NV parameters reported by Albrecht et al. [18], together with the common set of other required parameters outlined in the supplementary material. Yet, our model yields good agreement with the NV-MNP dimer based measurements of Schietinger et al. [3], as evident from the earlier comparisons based on Figure 2. It is worth mentioning that, like most fluorescent emitters, the NV center suffers in practice from non-unity quantum efficiencies (QEs) [47–49]. To obtain results that are independent of non-unity quantum efficiencies, we normalise the emission spectra relative to the isolated NV center emission.

### 3.2 Controlling NV emission using MNPs

**Figure 3:**
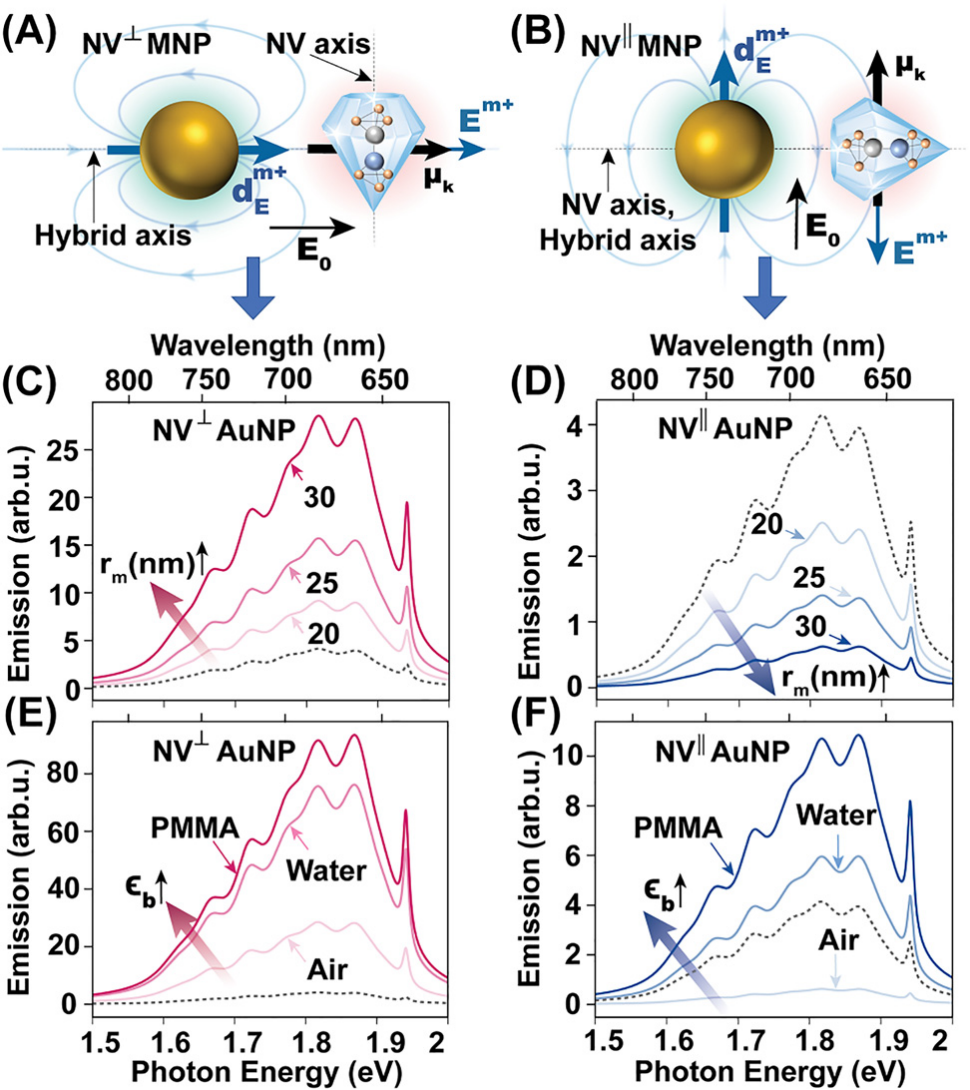
Controlling NV emission using a plasmonic nanoparticle. (A) Schematic diagram of the NV^⊥^MNP setup where the external field is polarized along the hybrid axis and NV dipoles are perpendicular to the MNP surface. (B) NV^‖^MNP setup where the external field is polarized perpendicular to the hybrid axis and NV dipoles are parallel to the MNP surface. Subplots (C) and (D) show the variation of total near-field NV emission of the NV^⊥^AuNP and NV^‖^AuNP dimers in air for different MNP radii at *R* = 38 nm. Subplots (E) and (F) depict the submerging medium dependence for NV^⊥^MNP and NV^‖^MNP dimers with *r*_m_ = 30 nm and *R* = 38 nm. Refractive index $n_{\text{b}}=\sqrt{\epsilon_{\text{b}}}\approx$ 1, 1.33, and 1.495 for air, water, and PMMA, respectively. Reference (dashed black) curves in subplots (C)–(F) show the isolated NV emission for the respective cases in air. All emission plots are normalized by the area of the respective reference curve.

We now investigate the possibility of controlling an NV center’s optical emission using a metal nanoparticle placed at nanoscale proximity. We focus on the NV^⊥^MNP and NV^‖^MNP setups schematically depicted in Figure 3(A) and (B), where the NV dipole orientations are perpendicular and parallel to the MNP surface, respectively. In the NV^⊥^MNP setup, both MNP and NV dipoles are oriented along the NV-MNP hybrid axis. Therefore, the NV center experiences an enhanced electric field due to the constructive superposition of the external field and the MNP dipole response field at the NV location, as observable in Figure 3(A). Conversely, in the NV^‖^MNP setup, the MNP dipole response field destructively interferes with the external field at the NV location as observable in Figure 3(B). This can result in suppression or enhancement of the NV emission intensity (compared to the isolated NV emission) depending on the strength of the MNP dipole response field, as we discuss below.

The impact of varying the MNP radius *r*_m_ is captured in Figure 3(C) and (D). In Figure 3(C), the NV emission intensity increases with *r*_m_ due to the accompanied enhancement of the MNP dipole response field that constructively superposes with the external field, in the NV^⊥^AuNP setup. Conversely, the NV emission intensity decreases with increasing *r*_m_ in the NV^‖^AuNP setup in Figure 3(D), which is indicative of the dominance of the external field over the MNP dipole response field, for all three values of *r*_m_ considered. Here, the reduction in emission intensity occurs due to the gradual increase of MNP dipole response field with increasing *r*_m_, causing the (screened) resultant field experienced by the NV center to decrease via destructive superposition.

The qualitative impact of reducing the NV-MNP center separation *R* for a fixed MNP radius *r*_m_ is similar to that of increasing *r*_m_ at a fixed separation *R* (and vice versa). This is because both increasing *r*_m_ and decreasing *R* result in increasing the positive frequency plasmonic field amplitude ***E*** ^m+^ experienced by the NV center in Figure 3(A) and (B). Further results and discussion can be found in the supplementary material.

We analyse the dependence of steady-state NV emission on the submerging medium permittivity in the presence of a metal nanoparticle in Figure 3(G) and (H). It can be observed that NV emission intensity is likely to enhance as the submerging medium permittivity increases, relative to the NV emission intensity observed for the respective dimer in air. The observed enhancement is partially attributable to the larger MNP dipole response field resulting from the increased dipolar polarizability of the MNP. Increasing the submerging medium permittivity relative to the emitter dielectric permittivity decreases the screening factor *ϵ*_effD_, resulting in an enhancement of the effective field (hence the effective Rabi frequencies) experienced by the NV center, contributing to the enhancement of steady state emission intensity. Furthermore, normalized emission rates for the NV center’s |*e*_0_⟩ → |*g*_*k*_⟩ transitions tend to increase with increasing medium permittivity, for both NV^⊥^AuNP and NV^‖^AuNP configurations, where stronger decay rate enhancements occur in the NV^⊥^AuNP case. The respective MNP polarizabilities and decay rate modification spectra can be found in the supplementary material.

We observe similar dependence of NV emission on MNP radius, center separation, and submerging medium permittivity in the presence of both large and small MNPs. Example results for small MNPs are included in the supplementary material.

**Figure 4:**
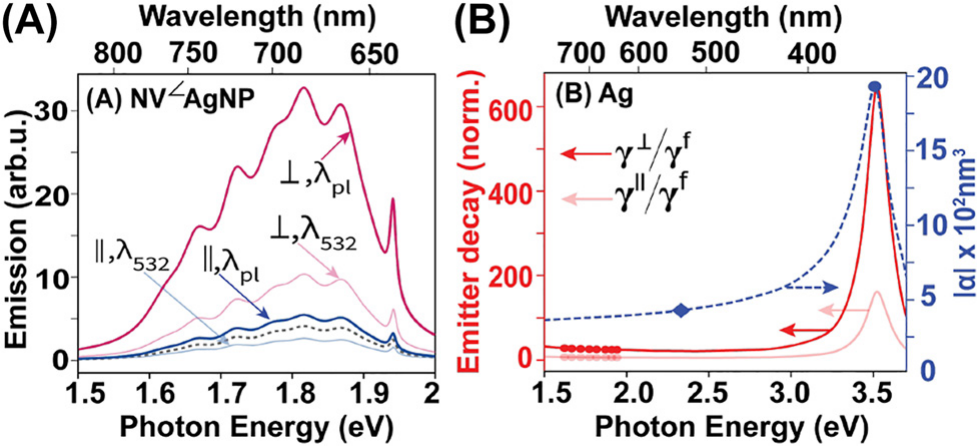
The impact of illuminating at plasmon resonance. (A) Total near-field emission of NV centers in NV^⊥^AgNP (*∠* → ⊥) and NV^‖^AgNP (*∠* → ‖) setups in air illuminated at the input wavelength 532 nm (*λ*_532_) and at the plasmon resonance wavelength of the adjacent AgNP (*λ*_pl_). The AgNP radius *r*_m_ = 7 nm and the NV-AgNP center separation *R* = 12 nm. The submerging medium is air. (B) The right *y*-axis shows the absolute polarizability |*α*| of the *r*_m_ = 7 nm AgNP in air. The blue diamond and circle depict the values of the input laser energies when illuminated at *λ*_532_ and *λ*_pl_, respectively. The estimated decay rates for generic emitters oriented radially (⊥) and tangentially (‖) to the surface of the same AgNP, normalized by the respective free-space decay rates *γ* ^f^(*ω*), are shown on the left *y*-axis. Red and pink circles depict the values at NV emission peaks, for NV^⊥^AgNP and NV^‖^AgNP orientations, respectively.

We finally explore the impact of illuminating NV-MNP hybrids with input radiation resonant and off-resonant with the plasmonic peak of the MNP in Figure 4. AuNPs typically possess plasmonic peak wavelengths close to 532 nm (see Figure 2). Therefore we consider a small (*r*_m_ = 7 nm) AgNP possessing a plasmonic peak far off-resonant from the commonly used 532 nm input laser for this analysis.

The NV center in the NV^⊥^AgNP setup experiences a significant emission enhancement compared to the respective 532 nm case when illuminated at the plasmon resonance wavelength of the AgNP. This is due to the larger plasmonic enhancement of the electric field experienced by the NV center. We also observe and an emission enhancement in the NV^‖^AgNP setup illuminated at the plasmonic peak of the AgNP, in contrast to the emission suppression observed for the same setup under 532 nm illumination. Such enhancement under destructive field superposition (see the NV^‖^MNP schematic in Figure 3(B)) indicates strong dominance of the MNP dipole response field over the external field at the NV location, resulting in the formation of a resultant field stronger than the external field, in the opposite direction.

### 3.3 Prospective applications and outlook

Our model demonstrates that the photoluminescence of NV centers can be greatly enhanced and controlled using nearby metal nanoparticles. NV-AuNP nanohybrids hold great potential in biomedical applications due to a multitude of reasons: both nanodiamonds and gold nanoparticles are largely inert, biocompatible, and their surfaces can be functionalized with a variety of targeting ligands [1, 8, 36, 50, 51]. The emission of the NV center readily resides in the near-infrared therapeutic window (650–900 nm) that exhibits high depths of tissue penetration, and the plasmon resonance [19] of AuNPs can be tuned to this region via structural elongation into nanorods [50]. Small nanodiamonds suitable for biomarking applications that are about an order of magnitude brighter than traditional red chromophores have been realized [1]. Our model demonstrates that their brightness can be further enhanced, retaining biocompatibility, using AuNPs.

Nanohybrids with relatively high quality factor plasmonic nanoparticles such as silver appear as powerful candidates for nanoscale optoelectronic devices in areas such as quantum information technology [1, 7, 52] and quantum sensing [53]. This is due to the large NV emission enhancements achievable by illuminating such hybrids at their plasmon resonance, and the high tunability of the resultant optical field experienced by the NV center.

Another promising future research avenue is to investigate the conditions under which the nonlinear (density matrix dependent) component of the NV Hamiltonian (1) outweighs the linear component. All of the systems encountered in this work are weakly nonlinear within our choices of metallic plasmonic materials and common submerging media, primarily due to the low coherence between the NV excited and ground states. Investigating and opening pathways to trigger strongly-nonlinear transients in the dynamics of the NV-plasmonic systems can lead to steady-states that depend on the initial state, a condition that can be exploited for enhanced sensing and for discerning NV-plasmonic configurations that cannot be distinguished otherwise.

## 4 Conclusions

We demonstrated that the optical interaction with a plasmonic metal nanoparticle (MNP) provides a robust handle to enhance and control the quantum sensing capabilities of a negatively charged nitrogen vacancy (NV) center. We have developed a rigorous theoretical model that successfully explains the existing experimental measurements of NV fluorescence, both in the presence and absence of an MNP. In essence, our model extends the existing bare NV optical model to capture the contributions of the ^3^ *E* vibronic levels and systematically accounts for both the local electric field and decay rate modifications induced by a proximal MNP. Our work reveals, for the first time, the ability to both suppress and enhance the NV fluorescence using metal nanoparticles in close proximity. The plasmonic effects can be controlled via the NV orientation, wavelength and polarization of the incoming light, submerging medium permittivity, and MNP type, size, and separation.

## Supplementary Material

Supplementary Material Details
