# Supplementary material for: NV-plasmonics: modifying optical emission of an NV− center via plasmonic metal nanoparticles
Source: Nanophotonics. 2022 Oct 19;11(21):4919–27. doi: 10.1515/nanoph-2022-0429 (PMC11501803; doi:10.1515/nanoph-2022-0429)
Supplement: Supplementary file 1 — Supplementary Material Details [file j_nanoph-2022-0429_suppl.pdf]

Supplementary Material
NV-plasmonics: modifying optical emission of an NV^- centre via plasmonic metal nanoparticles

Harini Hapuarachchi,[1,*] Francesco Campaioli,[1] and Jared H. Cole[1]

[1] *ARC Centre of Excellence in Exciton Science and Chemical and Quantum Physics, School of Science, RMIT University, Melbourne, 3001, Australia*

THEORETICAL MODEL

Model overview

In the context of optical interactions, we model the nitrogen-vacancy (NV) centre in diamond as a multi-level atom with $n+1$ ground states $\{|g_k\rangle\}$, $k \in \{0,\dots,n\}$, and two excited states $|e_0\rangle$ and $|e_1\rangle$. The number of phononic transitions from $|g_k\rangle$ to the zero phonon level $|g_0\rangle$ is k. The lowest energy excited level is denoted by $|e_0\rangle$, while we represent the higher vibronic levels above it by a phenomenologically defined single upper excited level $|e_1\rangle$ resonant with the frequency of incoming radiation. We consider excitation energies well above the zero-phonon transition energy of the NV centre throughout this work. We assume that the NV centre undergoes coherent transitions $|e_i\rangle \leftrightarrow |g_k\rangle$ ($i \in \{0,1\}$ and $k \in \{0,...,n\}$) upon the incidence of such coherent external radiation. A fast nonradiative (phononic) decay rate γ_{e} is defined between the two excited states, and dephasing is assumed to occur from both excited states to all ground states at rate γ_*. The incoherent optical emission transition corresponding to the zero phonon line (ZPL) is $|e_0\rangle \to |g_0\rangle$, whereas the incoherent emission transitions $|e_0\rangle \to |g_k\rangle$ ($k \neq 0$) contribute to the phononic side-bands of the NV optical emission spectrum. The nonradiative transitions between adjacent ground states $|g_k\rangle \to |g_{k-1}\rangle$ are characterized by the phononic decay rates $\gamma_{k,k-1}$ for $k \in \{1,...,n\}$. The schematic diagram of the NV centre can be found in Fig. 1(A) of the main text.

We assume that the NV centre undergoes optical dipole interactions with a spherical metal nanoparticle (MNP) of radius r_{m} placed at a nanoscale centre-separation R, upon the incidence of an external coherent electric field, $\boldsymbol{E} = E_0(e^{-i\omega_{\text{d}}t} + e^{i\omega_{\text{d}}t})\hat{\boldsymbol{e}}$. The unit vector along the field polarization direction is denoted by $\hat{\boldsymbol{e}}$, ω_{d} is the optical frequency, and t is time. Bold fonts denote vector quantities throughout the document. The total effective electric field experienced by the NV centre is altered due to the presence of the MNP. We denote the projection of this total effective field on the *NV dipolar plane* (introduced in the main text) by $\boldsymbol{E}_{\text{tot}}$. Both the diamond nanoparticle hosting the NV centre and the MNP are submerged in a medium of relative permittivity ϵ_{b}.

* harini.hapuarachchi@rmit.edu.au

Hamiltonian in the laboratory reference frame

Setting the energy of the zero-phonon ground state $|g_0\rangle$ to be zero, we can obtain the unperturbed Hamiltonian of the NV centre as follows:

$$\hat{H}_0 = \left(\sum_{k=0}^{n} \hbar\omega_k |g_k\rangle\langle g_k|\right) + \hbar\omega_{\text{z}}|e_0\rangle\langle e_0| + \hbar\omega_{\text{d}}|e_1\rangle\langle e_1|, \quad (1)$$

where $\hbar\omega_k$ is the energy of the kth ground state phononic level and $\hbar\omega_{\text{z}}$ is the ZPL energy. The perturbation Hamiltonian component arising due to the coherent dipolar interaction between the NV centre and the total effective field incident on it is obtainable by extending the treatment for two-level emitter-field interaction [1–4] as,

$$\hat{H}_{\text{int}} = -\sum_{k=0}^{n}\sum_{j=0}^{1} \left(|g_k\rangle\langle e_j| + |e_j\rangle\langle g_k|\right) \boldsymbol{\mu}_k \cdot \boldsymbol{E}_{\text{tot}}. \quad (2)$$

The dipole moment operator element corresponding to the transitions $|e_j\rangle \leftrightarrow |g_k\rangle$ is denoted by $\boldsymbol{\mu}_k$. Assuming that $\boldsymbol{\mu}_k$ aligns along $\boldsymbol{E}_{\text{tot}}$ we obtain,

$$\hat{H}_{\text{int}} = -E_{\text{tot}}\sum_{k=0}^{n}\sum_{j=0}^{1} \left(|g_k\rangle\langle e_j| + |e_j\rangle\langle g_k|\right) \mu_k. \quad (3)$$

The complete form of E_{tot} will be elaborated in a later section, for two special cases of interest. The total laboratory frame Hamiltonian of the NV centre under the influence of the externally incident field and the MNP can be obtained as,

$$\hat{H}_{\text{tot}} = \hat{H}_0 + \hat{H}_{\text{int}}. \quad (4)$$

Transformation into a rotating reference frame

To transform the laboratory (static) frame Hamiltonian into a rotating reference frame for the ease of computations, we define a unitary transformation operator,

$$\hat{U} = e^{i\hat{H}_1 t/\hbar}. \quad (5)$$

The Hamiltonian $\hat{H}_1 \approx \hbar\omega_{\text{d}}\left(|e_0\rangle\langle e_0| + |e_1\rangle\langle e_1|\right)$ is defined in the same eigenbasis as $\hat{H}_0$ with the ground states $\{|g_0\rangle,\dots,|g_n\rangle\}$ possessing eigenenergies ≈ 0 and the excited states $\{|e_0\rangle,|e_1\rangle\}$ possessing eigenenergies

$\approx \hbar\omega_{\rm d}$. Considering the exponential operator expansion of $e^{i\hat{H}_1 t/\hbar}$ and its adjoint where $|a\rangle$ represents any eigenstate of $\hat{H}_1$, we can obtain the following expressions,

$$e^{\pm i\hat{H}_1 t/\hbar}|a\rangle = e^{\pm i\omega_a t}|a\rangle, \tag{6a}$$

$$\langle a|e^{\mp i\hat{H}_1 t/\hbar} = e^{\mp i\omega_a t}\langle a|, \tag{6b}$$

where ω_a is the angular frequency of the eigenvalue corresponding to $|a\rangle$.

The laboratory reference frame Hamiltonian (4) can be transformed into the rotating reference frame with the following expression which can be derived using the Schrödinger equation [5],

$$\hat{H}_{\rm RF} = \hat{U}\left(\hat{H}_0 + \hat{H}_{\rm int}\right)\hat{U}^\dagger + i\hbar\dot{\hat{U}}\hat{U}^\dagger. \tag{7}$$

Simplifying (7) using (6a) and (6b), and applying the rotating wave approximation [6] (where we discard the fast oscillating terms that average out to zero in the population oscillation timescales of our concern) we obtain,

$$\hat{H}_{\rm RF} \approx \left(\sum_{k=0}^{n}\hbar\omega_k|g_k\rangle\langle g_k|\right) + \hbar(\omega_{\rm z}-\omega_{\rm d})|e_0\rangle\langle e_0| - \sum_{k=0}^{n}\sum_{j=0}^{1}\left(\hbar\Omega_k^{\rm r}|e_j\rangle\langle g_k| + \hbar\Omega_k^{\rm r*}|g_k\rangle\langle e_j|\right), \tag{8}$$

where $\Omega_k^{\rm r}$ is the effective Rabi frequency at which the NV centre's $|e_j\rangle \leftrightarrow |g_k\rangle$ transition is driven, and $\Omega_k^{\rm r*}$ is its complex conjugate. The kth Rabi frequency is related to the field incident on the NV dipolar plane such that $\hbar\Omega_k^{\rm r} = \mu_k\tilde{E}^+_{\rm tot}$, where $\tilde{E}^+_{\rm tot}$ denotes the slowly varying positive frequency amplitude of $E_{\rm tot} = \tilde{E}^+_{\rm tot}e^{-i\omega_{\rm d}t} + c.c.$.

Density matrix in the rotating reference frame

We first consider the density matrix of the NV centre in the laboratory reference frame, which represents a statistical ensemble of pure states $\{|\psi_l\rangle\}$ (in the same reference frame) occurring with probabilities $\{p_l\}$,

$$\hat{\rho}_{\rm L} = \sum_l p_l|\psi_l\rangle\langle\psi_l| = \begin{pmatrix} \rho_{g_0g_0} & \cdots & \rho^*_{g_ng_0} & \rho^*_{e_0g_0} & \rho^*_{e_1g_0} \\ \rho_{g_1g_0} & \cdots & \rho^*_{g_ng_1} & \rho^*_{e_0g_1} & \rho^*_{e_1g_1} \\ \vdots & \vdots & \vdots & \vdots & \vdots \\ \rho_{e_0g_0} & \cdots & \rho_{e_0g_n} & \rho_{e_0e_0} & \rho^*_{e_1e_0} \\ \rho_{e_1g_0} & \cdots & \rho_{e_1g_n} & \rho_{e_1e_0} & \rho_{e_1e_1} \end{pmatrix},$$

where $(^*)$ denotes the complex conjugate. Expanding the above density matrix using the outer products of the NV eigenbasis, we can write,

$$\hat{\rho}_{\rm L} = \hat{\rho}_{\rm diag} + \hat{\rho}_{\rm off}, \tag{9}$$

with

$$\hat{\rho}_{\rm diag} = \left(\sum_{k=0}^{n}\rho_{g_kg_k}|g_k\rangle\langle g_k|\right) + \rho_{e_0e_0}|e_0\rangle\langle e_0| + \rho_{e_1e_1}|e_1\rangle\langle e_1|,$$

$$\hat{\rho}_{\rm off} = \left(\sum_{k=0}^{n}\rho_{e_0g_k}|e_0\rangle\langle g_k| + \rho_{e_1g_k}|e_1\rangle\langle g_k|\right) + \rho_{e_1e_0}|e_1\rangle\langle e_0| + \left(\sum_{k=1}^{n}\sum_{h=0}^{k-1}\rho_{g_kg_h}|g_k\rangle\langle g_h|\right) + h.c.,$$

where $h.c.$ denotes the Hermitian conjugate of the entire matrix expression that precedes it.

We define a generic state vector transformed into the rotating reference frame $|\tilde{\psi}_l\rangle$ using the unitary operator (5) such that $|\tilde{\psi}_l\rangle = \hat{U}|\psi_l\rangle$, where $|\psi_l\rangle$ is the corresponding state vector in the laboratory (static) reference frame. This enables us to write the density matrix in the rotating reference frame as [6, 7],

$$\hat{\rho}_{\rm RF} = \sum_l p_l|\tilde{\psi}_l\rangle\langle\tilde{\psi}| = \sum_l p_l\hat{U}|\psi_l\rangle\langle\psi|\hat{U}^\dagger = \hat{U}\hat{\rho}_{\rm L}\hat{U}^\dagger. \tag{10}$$

The above equation can be simplified using (6a) and (6a) to obtain,

$$\hat{\rho}_{\rm RF} = \begin{pmatrix} \rho_{g_0g_0} & \cdots & \rho^*_{g_kg_0} & \cdots & \rho^*_{g_ng_0} & \tilde{\rho}^*_{e_0g_0} & \tilde{\rho}^*_{e_1g_0} \\ \vdots & \ddots & \vdots & \vdots & \vdots & \vdots & \vdots \\ \rho_{g_kg_0} & \cdots & \rho_{g_kg_k} & \cdots & \rho^*_{g_ng_k} & \tilde{\rho}^*_{e_0g_k} & \tilde{\rho}^*_{e_1g_k} \\ \vdots & \vdots & \vdots & \ddots & \vdots & \vdots & \vdots \\ \rho_{g_ng_0} & \cdots & \rho_{g_ng_k} & \cdots & \rho_{g_ng_n} & \tilde{\rho}^*_{e_0g_n} & \tilde{\rho}^*_{e_1g_n} \\ \tilde{\rho}_{e_0g_0} & \cdots & \tilde{\rho}_{e_0g_k} & \cdots & \tilde{\rho}_{e_0g_n} & \rho_{e_0e_0} & \rho^*_{e_1e_0} \\ \tilde{\rho}_{e_1g_0} & \cdots & \tilde{\rho}_{e_1g_k} & \cdots & \tilde{\rho}_{e_1g_n} & \rho_{e_1e_0} & \rho_{e_1e_1} \end{pmatrix}, \tag{11}$$

where the elements $\tilde{\rho}_{e_jg_k} = \tilde{\rho}^*_{g_ke_j}$ arise when factoring out the high-frequency time dependence of the coherences between the ground and excited states in the laboratory reference frame density matrix as,

$$\rho_{e_jg_k} = \tilde{\rho}_{e_jg_k}e^{-i\omega_{\rm d}t} \;\text{ for }\; k\in\{0,\ldots,n\}, j\in\{0,1\}. \tag{12}$$

All other elements in (11) remain the same as those of the laboratory reference frame density matrix $\rho_{\rm L}$.

Total effective field experienced by the NV centre

Due to each $|e_j\rangle \leftrightarrow |g_k\rangle$ transition in the NV centre, a classically expected oscillating dipole moment of the following form is assumed to be induced (extending the two-level atom based procedure in references [2, 4, 8, 9]),

$$\langle\hat{d}_{jk}\rangle = d_{jk} = \mu_k\left(\rho_{g_ke_j} + \rho_{e_jg_k}\right), \tag{13}$$

where $\hat{d}_{jk}$ is the respective NV dipole moment operator. The positive frequency components of the dipole moments induced in the MNP in response to each of

these oscillating dipole moments in the NV centre can be obtained as [9],

$$d_{jk}^{\mathrm{m}+} = \frac{s_\alpha \alpha(\omega_\mathrm{d}) \mu_k \tilde{\rho}_{e_j g_k} e^{-i\omega_\mathrm{d} t}}{\epsilon_\mathrm{effD} R^3}, \quad (14)$$

where $\alpha(\omega_\mathrm{d})$ is the polarizability of the MNP at angular frequency ω_d, the complete form of which will be presented later. The orientation parameter $s_\alpha = 2$ when both NV and MNP dipoles are aligned along the NV-MNP hybrid axis with the NV dipoles (d_{jk}) aligned perpendicular to the MNP surface (NV$^\perp$MNP). The parameter $s_\alpha = -1$ when both NV and MNP dipoles are aligned perpendicular to the NV-MNP hybrid axis with the NV dipoles aligned parallel to the MNP surface (NV$^\parallel$MNP). The optical field screening experienced by the NV centre due to the emitter material (diamond) with relative permittivity ϵ_D is incorporated using the following screening factor [2];

$$\epsilon_\mathrm{effD} = (2\epsilon_\mathrm{b} + \epsilon_\mathrm{D})/(3\epsilon_\mathrm{b}). \quad (15)$$

The positive frequency component of the dipole moment directly induced in the MNP due to the external field, along the same direction, is given by [10],

$$d_\mathrm{E}^{\mathrm{m}+} = (4\pi\epsilon_0\epsilon_\mathrm{b})\alpha(\omega_\mathrm{d}) E_0 e^{-i\omega_\mathrm{d} t}, \quad (16)$$

where ϵ_0 is the absolute permittivity of free-space. The positive frequency component of the total dipole moment induced in the MNP due to the external field and the NV transition dipoles can be obtained using (14) and (16) as,

$$d_\mathrm{tot}^{\mathrm{m}+} = d_\mathrm{E}^{\mathrm{m}+} + \sum_{k=0}^{n} \sum_{j=0}^{1} d_{jk}^{\mathrm{m}+}. \quad (17)$$

We can obtain the positive frequency component of the total effective field incident on the NV centre as the sum of the externally incident field and the total dipole response field of the MNP screened by the diamond lattice (extending the procedure for a two-level emitter implemented in [3] and [9]) as,

$$E_\mathrm{tot}^{+} = \frac{1}{\epsilon_\mathrm{effD}} \left\{ E_0 + \frac{s_\alpha d_\mathrm{tot}^{\mathrm{m}+}}{(4\pi\epsilon_0\epsilon_\mathrm{b}) R^3} \right\} e^{-i\omega_\mathrm{d} t}. \quad (18)$$

Expanding the above equation, we can obtain the complete form of the electric field experienced by an NV centre in NV$^\perp$MNP or NV$^\parallel$MNP orientation as,

$$E_\mathrm{tot} = \frac{1}{\epsilon_\mathrm{effD}} \left\{ E_0 + \frac{s_\alpha \alpha(\omega_\mathrm{d}) E_0}{R^3} + \frac{s_\alpha^2 \alpha(\omega_\mathrm{d})}{(4\pi\epsilon_0\epsilon_\mathrm{b})\epsilon_\mathrm{effD} R^6} \sum_{j=0}^{1} \sum_{k=0}^{n} \left(\mu_k \tilde{\rho}_{e_j g_k} \right) \right\} e^{-i\omega_\mathrm{d} t} + c.c., \quad (19)$$

where *c.c.* denotes the complex conjugate of the entire preceding expression.

We can express the positive frequency amplitude of the above field in terms of the Rabi frequency (or its decomposition) for any $|e_j\rangle \leftrightarrow |g_k\rangle$ transition as follows,

$$\tilde{E}_\mathrm{tot}^{+} = \frac{\hbar}{\mu_k} \Omega_k^\mathrm{r} = \frac{\hbar}{\mu_k} \left\{ \Omega_k + \eta_k \sum_{j=0}^{1} \sum_{l=0}^{n} \left(\mu_l \tilde{\rho}_{e_j g_l} \right) \right\}, \quad (20)$$

where the Ω_k denotes the Rabi frequency in the absence of coherences (when all $\tilde{\rho}_{e_j g_k} = 0$) and η_k is the NV self-interaction coefficient obtainable as,

$$\Omega_k = \frac{\mu_k E_0}{\hbar \epsilon_\mathrm{effD}} \left\{ 1 + \frac{s_\alpha \alpha(\omega_\mathrm{d})}{R^3} \right\}. \quad (21a)$$

$$\eta_k = \frac{\mu_k s_\alpha^2 \alpha(\omega_\mathrm{d})}{(4\pi\epsilon_0\epsilon_\mathrm{b})\hbar \epsilon_\mathrm{effD}^2 R^6}. \quad (21b)$$

In the absence of the MNP (when $r_\mathrm{m} \to 0$ or $R \to \infty$),

$$\Omega_k \to \Omega_k^0 = \frac{\mu_k E_0}{\hbar \epsilon_\mathrm{effD}}, \quad (22a)$$

$$\eta_k \to \eta_k^0 = 0. \quad (22b)$$

The above approach was inspired by a formalism established in the literature in the context of quantum dot-MNP interaction [3, 9, 11–13]. Here, we have adapted and extended it to the context of NV-MNP optical interaction, for the first time.

MNP polarization

We now focus on the optical response of the metal nanoparticle, incorporated into our model using its dipolar polarizability at angular frequency ω_d, $\alpha(\omega_\mathrm{d})$. The extensively used format of the MNP polarizability in the literature arises from the solution of Laplace equation for the electric potential ($\nabla^2\phi = 0$) for a homogenous, isotropic sphere embedded in an isotropic, non-absorbing submerging medium upon the incidence of a spatially static (temporally oscillating) electric field [10]. The resulting polarizability is given by,

$$\alpha_\mathrm{L}(\omega_\mathrm{d}) = r_\mathrm{m}^3 \frac{\epsilon_\mathrm{m}(\omega_\mathrm{d}) - \epsilon_\mathrm{b}}{\epsilon_\mathrm{m}(\omega_\mathrm{d}) + 2\epsilon_\mathrm{b}}, \quad (23)$$

where $\epsilon_{\rm m}(\omega_{\rm d})$ is the spatially constant relative permittivity of the metal at frequency $\omega_{\rm d}$. It has been shown that this lowest-order (dipolar) full scattering problem under the *quasi-static* and local response approximations (LRA) [14] describes the optical properties of nanoparticles of dimensions below 100 nm adequately for many purposes [10]. The quasi-static local dipolar polarizability in equation (23) has been further improved in the contexts of large and small MNPs as follows;

Large MNPs

For large MNPs (for example, with diameters ~ 80 nm [15]), equation (23) which models the MNP as an ideal dipole does not satisfy the optical theorem (energy conservation) [15, 16]. This apparent paradox has been overcome by taking the finite-size effects into account, which leads to an effective dipolar polarizability [16–18],

$$\alpha_{\rm eff}(\omega_{\rm d}) = \frac{\alpha_{\rm L}(\omega_{\rm d})}{\left[1 - \frac{2ik_{\rm b}^3}{3}\alpha_{\rm L}(\omega_{\rm d})\right]}. \tag{24}$$

Wavenumber of the non-absorbing submerging medium is $k_{\rm b} = n_{\rm b}k$, where $n_{\rm b} = \sqrt{\epsilon_{\rm b}}$ is the refractive index of the (non-magnetic) medium and $k = \omega_{\rm d}/c$ is the free-space wavenumber (c is the speed of light). The effective polarizability $\alpha_{\rm eff}$ accounts for the radiative reaction (impact of finite size) of the MNP which microscopically originates from radiation emitted by the charge oscillations induced inside the nanoparticle by the external field [16].

Small MNPs

Size dependent plasmon resonance shifts and linewidth broadening phenomena that cannot be captured using the local optical polarizability (23) has been observed in recent plasmonic experiments involving MNPs of diameter $\lesssim$ 20 nm [14]. These effects arise due to a nanoscale physical mechanism beyond classical electrodynamics known as the nonlocal response [19]. The *generalized nonlocal optical response* (GNOR) theory that accounts for both electron pressure and electron diffusion effects in such small MNPs was recently introduced by Mortensen and Raza *et al* [14, 20]. The GNOR theory introduces a nonlocal correction ($\delta_{\rm NL}$) to the LRA based dipolar polarizability in (23) as follows [14],

$$\alpha_{\rm NL}(\omega_{\rm d}) = r_{\rm m}^3 \frac{\epsilon_{\rm m}(\omega_{\rm d}) - \epsilon_{\rm b}\left[1 + \delta_{\rm NL}(\omega_{\rm d})\right]}{\epsilon_{\rm m}(\omega_{\rm d}) + 2\epsilon_b\left[1 + \delta_{\rm NL}(\omega_{\rm d})\right]}. \tag{25}$$

The nonlocal correction is given by,

$$\delta_{\rm NL}(\omega_{\rm d}) = \frac{\left[\epsilon_{\rm m}(\omega_{\rm d}) - \epsilon_{\rm core}(\omega_{\rm d})\right] j_1(k_{\rm NL}(\omega_{\rm d})r_m)}{\epsilon_{\rm core}(\omega_{\rm d})k_{\rm NL}(\omega_{\rm d})r_m j_1'(k_{\rm NL}(\omega_{\rm d})r_m)}, \tag{26}$$

where $k_{\rm NL}$ is the longitudinal wavenumber in the GNOR model obtainable as [14],

$$k_{\rm NL}^2(\omega_{\rm d}) = \frac{\omega_{\rm d}(\omega_{\rm d} + i\Gamma_{\rm m})\epsilon_{\rm m}(\omega_{\rm d})}{\epsilon_{\rm core}(\omega_{\rm d})\left[\beta^2 + D_{\rm m}(\Gamma_{\rm m} - i\omega_{\rm d})\right]}. \tag{27}$$

In the above equations, the response of bound electrons, bulk plasmon damping rate, and electron diffusion constant of the MNP are denoted by $\epsilon_{\rm core}(\omega_{\rm d})$, $\Gamma_{\rm m}$, and $D_{\rm m}$, respectively. In the high-frequency limit where $\omega_{\rm d} \gg \Gamma_{\rm m}$, $\beta^2 = (3/5)v_{\rm F}^2$ where $v_{\rm F}$ denotes the Fermi velocity of the MNP.

We use $\alpha(\omega_{\rm d}) = \alpha_{\rm eff}(\omega_{\rm d})$ for large nanoparticles, and $\alpha(\omega_{\rm d}) = \alpha_{\rm NL}(\omega_{\rm d})$ for small nanoparticles when generating our results using the equations presented in earlier sections.

MNP-induced NV decay rate modifications

It has been shown that the rate of radiative emission is not an inherent property of emitters such as quantum dots and NV centres [21, 22]. This rate is rather determined by the interaction between the emitter and its local electromagnetic environment [22], which in this case comprises the metal nanoparticle. To capture such modifications we incorporate the equations outlined in the following sections into our model.

Large MNPs

To estimate the decay rate modification experienced by the kth NV emission transition for the cases of normal ($\perp$) and tangential ($\|$) emitter dipole orientations with respect to the surface of an adjacent large MNP, we use the following equations derived by Carminati *et al.* where the MNP is treated within the dipole approximation, while accounting for the finite size effects [18],

$$\frac{\gamma_k^{\perp}}{\gamma_k^{\rm f}} \approx n_{\rm b}\left\{1 + 6k_{\rm b}^3 {\rm Im}\left[\alpha_{\rm eff}(\omega)e^{2ik_{\rm b}R}\left(\frac{-1}{(k_{\rm b}R)^4} + \frac{2}{i(k_{\rm b}R)^5} + \frac{1}{(k_{\rm b}R)^6}\right)\right]\right\}, \tag{28a}$$

$$\frac{\gamma_k^{\|}}{\gamma_k^{\rm f}} \approx n_{\rm b}\left\{1 + \frac{3}{2}k_{\rm b}^3 {\rm Im}\left[\alpha_{\rm eff}(\omega)e^{2ik_{\rm b}R}\left(\frac{1}{(k_{\rm b}R)^2} - \frac{2}{i(k_{\rm b}R)^3} - \frac{3}{(k_{\rm b}R)^4} + \frac{2}{i(k_{\rm b}R)^5} + \frac{1}{(k_{\rm b}R)^6}\right)\right]\right\}. \tag{28b}$$

The above equations consider an emitter in a medium with refractive index $n_{\rm b}$. The free-space decay rate of

the emission transition considered is denoted by γ_k^{f}, and ω is the angular frequency of the same transition.

Small MNPs

For small MNPs submerged in low refractive index media $\alpha_{\mathrm{eff}} \approx \alpha_{\mathrm{L}}$. Using equations (23) and (24) together with (interpolated) MNP permittivity data from Johnson and Christy's tabulations [23], we verified this claim for small gold nanoparticles with radii $r_{\mathrm{m}} \lesssim 7\,\mathrm{nm}$ in media with refractive index $n_{\mathrm{b}} \lesssim 1.5$, and for small silver nanoparticles with $r_{\mathrm{m}} \lesssim 7\,\mathrm{nm}$ in air. By incorporating the GNOR based nonlocal correction to the decay rate modification equations derived by des Francs *et al.* [16, 17] for $k_{\mathrm{b}}R \ll 1$ we obtain,

$$\frac{\gamma_k^{\perp}}{\gamma_k^{\mathrm{f}}} \approx \frac{6n_{\mathrm{b}}\mathrm{Im}\left[\alpha_{\mathrm{NL}}(\omega)\right]}{k_{\mathrm{b}}^3R^6}, \tag{29a}$$

$$\frac{\gamma_k^{\parallel}}{\gamma_k^{\mathrm{f}}} \approx \frac{3n_{\mathrm{b}}\mathrm{Im}\left[\alpha_{\mathrm{NL}}(\omega)\right]}{2k_{\mathrm{b}}^3R^6}. \tag{29b}$$

We recently used the above equations to successfully describe the decay rate modification of quantum dots near small MNPs, at $\sim$10 nm surface separations [24].

NV centre as an open quantum system

The Hamiltonian of the NV centre optically coupled to the externally incident field and the MNP dipole response field represents a closed quantum system where the impact of the environment (bath) is yet to be taken into account. It couples with the environment resulting in an open quantum system with irreversible dynamics. We estimate the evolution of the density matrix of an open quantum system weakly coupled to a Markovian (memoryless) bath using the following master equation [25],

$$\dot{\hat{\rho}}_{\mathrm{RF}} = -\frac{i}{\hbar}[\hat{H}_{\mathrm{RF}}, \hat{\rho}_{\mathrm{RF}}] + \sum_x \Gamma_x[\hat{L}_x\hat{\rho}_{\mathrm{RF}}\hat{L}_x^{\dagger} - \frac{1}{2}\{\hat{L}_x^{\dagger}\hat{L}_x, \hat{\rho}_{\mathrm{RF}}\}], \tag{30}$$

where $\hat{L}_x$ is the Lindblad or collapse operator corresponding to the xth decoherence channel with characteristic decoherence rate Γ_x. The mathematical operators $[\cdot,\cdot]$ and $\{\cdot,\cdot\}$ denote the commutator and anti-commutator of the operands. The decoherence channels and the corresponding rates considered in our extended NV centre model schematically depicted in Fig. 1(A) of the main text are as follows:

For each optical decay transition $|e_0\rangle \to |g_k\rangle$:
$\Gamma_x = \gamma_k$, for $k \in \{0, \ldots, n\}$
$\hat{L}_x = \hat{\sigma}_k = |g_k\rangle\langle e_0|$

For each phononic decay transition $|g_k\rangle \to |g_{k-1}\rangle$:
$\Gamma_x = \gamma_{k,k-1}$, for $k \in \{1, \ldots, n\}$
$\hat{L}_x = |g_{k-1}\rangle\langle g_k|$

Nonradiative decay in the excited state $|e_1\rangle \to |e_0\rangle$:
$\Gamma_x = \gamma_{\mathrm{e}}$
$\hat{L}_x = |e_0\rangle\langle e_1|$

Dephasing from excited to ground states:
$\Gamma_x = \gamma_*$
$\hat{L}_x = |e_0\rangle\langle e_0| + |e_1\rangle\langle e_1|$

Emission intensity spectrum

This section outlines how we can utilize the steady state density matrix obtainable by solving (30) to estimate the emission intensity spectrum of the NV centre. The free-space fluorescence or emitted power spectrum $S_f(\omega)$ of a generic two-level emitter in a stationary state can be calculated using its emission correlation function in the following form [25–28],

$$S_f(\omega) = f(\boldsymbol{r})\int_{-\infty}^{\infty} d\tau e^{-i\omega\tau}\langle\hat{\sigma}^{\dagger}(\tau)\hat{\sigma}(0)\rangle_{\mathrm{ss}}, \tag{31}$$

employing the homogeneity in time of the stationary correlation function. In the above equation, $\hat{\sigma} = |g\rangle\langle e|$ denotes the emission operator from an excited state $|e\rangle$ to a ground state $|g\rangle$, ω denotes angular frequency, and $\langle\cdot\rangle_{\mathrm{ss}}$ is the expectation calculated using the steady state density matrix. The coefficient $f(\boldsymbol{r})$ is a geometrical factor defined such that $f(\boldsymbol{r}) \propto \omega_{\mathrm{eg}}\gamma$, where ω_{eg} and γ denote the emitter resonance frequency and the free-space decay rate, respectively. The vector $\boldsymbol{r}$ measures positions with respect to an origin at the location of the emitter [26]. We can estimate the photon emission intensity by normalizing the power spectrum in (31) by the emitted photon energy $\approx \hbar\omega_{\mathrm{eg}}$. Assuming emission behaviour analogous to the above generic two-level case for each $|e_0\rangle \to |g_k\rangle$ transition, and summing up the intensity spectra resulting from all such transitions, we estimate the total photon emission intensity spectrum of the NV centre as,

$$I_{\mathrm{tot}}(\omega) \propto \sum_{k=0}^{n}\gamma_k\int_{-\infty}^{\infty} d\tau e^{-i\omega\tau}\langle\hat{\sigma}_k^{\dagger}(\tau)\hat{\sigma}_k(0)\rangle_{\mathrm{ss}}, \tag{32}$$

where $\hat{\sigma}_k = |g_k\rangle\langle e_0|$. This expression is validated by comparison against the experimentally observed NV emission intensity spectra in Fig. 1(C) in the main text.

NUMERICAL IMPLEMENTATION

The piecewise superoperator method

To numerically simulate the NV centre's emission behaviour, we first need to solve the master equation (30)

for its steady state density matrix. The common procedure used in the literature [2, 29] when numerically solving similar emitter-MNP systems is the element-wise decomposition of the master equation into a set of coupled differential equations followed by the use of differential equation solvers such as *Runge-Kutta* [30] implementations readily available in Matlab and Python. Due to the evolution timescales involved in our problem, such procedures take extremely long computational times to reach the steady state. To solve the problem in hand within much shorter computational times, we propose the following piecewise superoperator method.

We first decompose the Hamiltonian $\hat{H}_{\rm RF}$ in (8) experiencing the Rabi frequencies in (20) into linear and nonlinear components as $\hat{H}_{\rm RF} \approx \hat{H}_{\rm lin} + \hat{H}_{\rm nl}$, where the linear part is,

$$H_{\rm lin} = \left(\sum_{k=0}^{n} \hbar\omega_k |g_k\rangle\langle g_k|\right) + \hbar(\omega_{\rm z} - \omega_{\rm d})|e_0\rangle\langle e_0| - \sum_{k=0}^{n}\sum_{j=0}^{1} \hbar\Omega_k |e_j\rangle\langle g_k| + \hbar\Omega_k^* |g_k\rangle\langle e_j|. \quad (33)$$

The nonlinear part is,

$$H_{\rm nl} = -\sum_{k=0}^{n}\sum_{j=0}^{1} \hbar {\rm nl}_k^{\rm coeff} |e_j\rangle\langle g_k| + \hbar {\rm nl}_k^{\rm coeff*} |g_k\rangle\langle e_j|, \quad (34)$$

where $(^*)$ denotes the complex conjugate and,

$${\rm nl}_k^{\rm coeff} = \eta_k \sum_{j=0}^{1}\sum_{l=0}^{n} \left(\mu_l \tilde{\rho}_{e_j g_l}\right). \quad (35)$$

Then we judiciously insert the identity operator $\hat{I}$ into the master equation (30) as,

$$\hat{I}\dot{\hat{\rho}}\hat{I} = -\frac{i}{\hbar}(\hat{H}_{\rm RF}\hat{\rho}_{\rm RF}\hat{I} - \hat{I}\hat{\rho}_{\rm RF}\hat{H}_{\rm RF}) + \sum_x \Gamma_x [L_x \hat{\rho}_{\rm RF} L_x^\dagger - \frac{1}{2}(L_x^\dagger L_x \hat{\rho}_{\rm RF}\hat{I} + \hat{I}\hat{\rho}_{\rm RF} L_x^\dagger L_x)], \quad (36)$$

Using the following vector identity in the column-ordered form [31, 32],

$${\rm vec}(\hat{A}\hat{X}\hat{B}) = (\hat{B}^T \otimes \hat{A}){\rm vec}(\hat{X}), \quad (37)$$

we can obtain the following superoperator form of the master equation,

$$\dot{\vec{\rho}}_{\rm RF} = \hat{\mathcal{L}}\vec{\rho}_{\rm RF}. \quad (38)$$

The vectorised density matrix in the column-ordered form is given by $\vec{\rho}_{\rm RF} = {\rm vec}(\rho_{\rm RF})$, and $\hat{\mathcal{L}} = \hat{\mathcal{L}}_{\rm lin} + \hat{\mathcal{L}}_{\rm nl}$ is the superoperator (Liouvillian) decomposed into its linear and nonlinear components, where the linear part is,

$$\hat{\mathcal{L}}_{\rm lin} = -\frac{i}{\hbar}\left[\hat{I} \otimes \hat{H}_{\rm lin} - \hat{H}_{\rm lin}^T \otimes \hat{I}\right] + \sum_x \Gamma_x \left\{(L_x^* \otimes L_x) - \frac{1}{2}\left[(L_x^\dagger L_x)^{\rm T} \otimes \hat{I} + (\hat{I} \otimes L_x^\dagger L_x)\right]\right\}, \quad (39)$$

and the nonlinear part (that depends on elements of $\hat{\rho}_{\rm RF}$) is given by,

$$\hat{\mathcal{L}}_{\rm nl}(\hat{\rho}_{\rm RF}) = -\frac{i}{\hbar}\left[\hat{I} \otimes \hat{H}_{\rm nl} - \hat{H}_{\rm nl}^T \otimes \hat{I}\right]. \quad (40)$$

In the absence of non-linearities (when $\hat{\mathcal{L}} = \hat{\mathcal{L}}_{\rm lin}$ is independent of both density matrix elements and time), the solution to (38) takes the form,

$$\vec{\rho}_{\rm RF}(t) = e^{\hat{\mathcal{L}}_{\rm lin} t}\vec{\rho}_{\rm RF}(0). \quad (41)$$

That is, $e^{\hat{\mathcal{L}}_{\rm lin} t}$ propagates a linear system from the initial state to the state at time t. To solve the nonlinear problem in hand, we subdivide the total propagation timescale into small (adaptive) time-steps δt within each of which the system is assumed to exhibit piecewise linear behaviour of the form,

$$\vec{\rho}_{\rm RF}(t + \delta t) \approx e^{\hat{\mathcal{L}}(t)\delta t}\vec{\rho}_{\rm RF}(t), \quad \text{where,} \quad \hat{\mathcal{L}}(t) = \hat{\mathcal{L}}_{\rm lin} + \hat{\mathcal{L}}_{\rm nl}(\hat{\rho}_{\rm RF}(t)). \quad (42)$$

The nonlinear piecewise evolution can be implemented using the *liouvillian*() function of the Quantum Toolbox in Python (QuTiP) [33] as outlined in Algorithm 1.

Algorithm 1: Piecewise superoperator evolution

input:
Final times for evolution regions, $\text{tf}_{\rm list} = [T_1, T_2, T_3]$
Adaptive time-steps, $\delta t_{\rm list} = [\delta t_1, \delta t_2, \delta t_3]$
Linear Hamiltonian, $\hat{H}_{\rm lin}$
List of collapse operators with rates, $c_{\rm ops} = \{\sqrt{\Gamma_x}\hat{L}_x\}$
List of η_k values, $\eta_{\rm list} = [\eta_0, \ldots, \eta_n]$
List of μ_l values, $\mu_{\rm list} = [\mu_0, \ldots, \mu_n]$
Initial state, $\hat{\rho}_0 = \hat{\rho}_{\rm RF}(0)$
Initial time, $t_0 = 0$

output:
List of evolution times, $t_{\rm list}$
List of evolved states, $\hat{\rho}_{\rm list}$

begin:
Initialize empty lists $t_{\rm list}, \hat{\rho}_{\rm list}$
Initialize current state and time $\hat{\rho}_{\rm RF} = \hat{\rho}_0$, $t = t_0$
$t_{\rm list}$.append(t)
$\hat{\rho}_{\rm list}$.append($\hat{\rho}_{\rm RF}$)
$\hat{\mathcal{L}}_{\rm lin} = liouvillian(\hat{H}_{\rm lin}, c_{\rm ops})$

for each T_i in $\text{tf}_{\rm list}$:
  while $t < \text{tf}_{\rm list}[i]$:
    $t = t + \delta t_i$
    Build $\hat{H}_{\rm nl}$ using $\eta_{\rm list}$, $\mu_{\rm list}$ and current $\hat{\rho}_{\rm RF}$
    $\hat{\mathcal{L}} = \hat{\mathcal{L}}_{\rm lin} + liouvillian(\hat{H}_{\rm nl}, [\,])$
    $\hat{\mathcal{P}}_i = e^{\hat{\mathcal{L}}\delta t_i}$
    vectorized($\hat{\rho}_{\rm RF}$) = $\hat{\mathcal{P}}_i \times$ vectorized($\hat{\rho}_{\rm RF}$)
    $\hat{\rho}_{\rm RF}$ = *vector_to_operator* (vectorized($\hat{\rho}_{\rm RF}$))
    $t_{\rm list}$.append(t)
    $\hat{\rho}_{\rm list}$.append($\hat{\rho}_{\rm RF}$)
  end
end

k	A_k (arb.u)	$\hbar\omega_k$ (meV)	$\gamma_k^{\rm f}$ (MHz)	$\gamma_{k,k-1}$ (THz)
0	1520	0	0.69	-
1	5260	31.8	2.42	85
2	18600	70.3	8.57	82
3	16400	124	7.57	79
4	14000	168	6.46	88
5	9180	221	4.23	65
6	6570	275	3.03	71
7	3270	319	1.51	86

TABLE I. Room temperature NV parameters from [34].

When implementing the algorithm presented above, we decomposed the total evolution time (≈300 ns) into three regions with final times $[T_1 = 10^{-4}T, T_2 = 10^{-3}T, T_3 = 10^3 T]$, and time steps $[\delta t_1 = 10^{-6}T, \delta t_2 = 10^{-5}T, \delta t_3 = 5T]$ for the three regions, respectively. A characteristic time $T = 2\pi/|\Omega_0^{\rm f}|$ was defined, where $\Omega_0^{\rm f} = \mu_0 E_0/(\hbar\epsilon_{\rm effD})$ in air, obtained using parameters outlined in the next section.

The density matrix evolution resulting from the proposed piecewise superoperator method was verified against the results of (4-5th order) *Runge Kutta* implementation in Python for the parameter region of our concern. The newly proposed method together with the carefully chosen adaptive time-steps reduces the computational time taken by a single evolution of (30) to reach the steady state from several days on a supercomputer (for the conventional *Runge-Kutta* solving) to a few seconds on a generic computer.

Metal	$\hbar\Gamma_{\rm m}$(eV)	$\hbar\omega_{\rm p}$(eV)	$v_{\rm F}(10^6{\rm m\,s^{-1}})$	$D_{\rm m}$ $(10^{-4}{\rm m^2\,s^{-1}})$
Au	0.071	9.02	1.39	8.62
Ag	0.025	8.99	1.39	9.62

TABLE II. Metal parameters from [14].

Common parameters used

Throughout this work, we use the set of NV parameters obtained by Albrecht *et al.* in [34] for a single NV centre in a nanodiamond at room temperature. This has been done by fitting the NV emission intensity spectrum in air with 8 Lorentzian lines ($n = 7$) with scaled amplitudes. These amplitudes A_k, phonon energies $\hbar\omega_k$, free-space decay rates $\gamma_k^{\rm f}$, and phonon decay rates $\gamma_{k,k-1}$ are presented in Table I. We modify the free-space decay rates using equations (28) or (29) to obtain γ_k of the NV centre in the presence of an MNP, as discussed earlier. The energy of the NV zero-phonon line $\hbar\omega_{\rm z} = 1.941\,{\rm eV}$ [34], and the dephasing rate between the ground and excited states $\gamma_* = 15\,{\rm THz}$ [34] is used for all transitions.

In [34], each $\gamma_k^{\rm f}$ is obtained by scaling the total decay rate $\gamma_{\rm tot} \sim 1/29\,{\rm ns}$ (for NV in nanodiamond) such that $\gamma_k^{\rm f} = \varepsilon_k \cdot \gamma_{\rm tot}$ where $\varepsilon_k = A_k / \sum_k A_k$. It is noteworthy that the effective excited state lifetime in air (or free-space) $1/\sum \gamma_k^{\rm f}$ resulting from the decay rates reported by Albrecht *et al.* in [34] is quite close to the optical excited state (^3E) lifetime measurement for a single NV centre in nanodiamond $\sim 25\,{\rm ns}$ reported by Beveratos *et al.* in [35]. It is observable that optical excited state (^3E) lifetimes in nanodiamond crystals that are considerably smaller than the fluorescence wavelength approximately double in comparison to NV centres in bulk diamond. This change is has been attributed to the reduction of the radiative emission rate induced by the decrease of the effective refractive index of the medium surrounding the NV centre [36].

The absolute angle-averaged optical dipole moment element for the NV centre is obtained from [37] as $\mu_{e\leftrightarrow g} \sim 5.2\,{\rm D}$. In our model, we assume $5.2\,{\rm D} \sim \mu_0$ (the dipole moment element that corresponds to the $|e_j\rangle \leftrightarrow |g_0\rangle$ transition for both $j = 0$ and 1). We then estimate the scaled dipole moment elements for other optical transitions $|e_j\rangle \leftrightarrow |g_k\rangle$ as $\mu_k = \sqrt{\varepsilon_k/\varepsilon_0}\mu_0$, such that $\gamma_k^{\rm f} \propto |\mu_k|^2$ for each transition (as required by both Fermi's golden rule and Einstein A coefficient for a generic emitter [1, 26, 38]).

We estimate the total nonradiative decay rate between excited levels as $\gamma_{\rm e} \sim \frac{1}{n}\left(\sum_k \gamma_{k,k-1}\right) * n_{\rm e}$, where $n_{\rm e}$ is the expected number of phonons between $|e_1\rangle$ and $|e_0\rangle$ obtained assuming that the average energy of a phonon in the ground and excited states are similar.

The positive frequency amplitude of the externally incident field is $E_0 = 30 \times 10^4\,{\rm V\,m^{-1}}$ (such that the resulting Rabi frequencies are in the GHz range). The refractive indices of air, water, and PMMA were taken as $n_{\rm b} \approx 1$, $n_{\rm b} \approx 1.33$, and $n_{\rm b} \approx 1.495$, respectively. The refractive index of diamond, $n_{\rm D} \approx 2.4$.

Dielectric permittivity of the MNP $\epsilon_{\rm m}$ was obtained by interpolating the tabulations by Johnson and Christy [1] for both Au and Ag. The bound electron response for a given angular frequency ω was obtained using the relationship, $\epsilon_{\rm core}(\omega) = \epsilon_{\rm m}(\omega) + \omega_{\rm p}^2/(\omega^2 + i\omega\Gamma_{\rm m})$ [14], where $\omega_{\rm p}$ is the bulk plasma frequency of the metal. Additionally, the parameters in Table II obtained from [14] were used when modelling the Au and AgNPs.

The values of any other parameters used (MNP radius $r_{\rm m}$, NV-MNP centre separation R, the submerging medium considered, and the orientation parameter s_α) will be presented alongside each set of results, separately.

Generation of sample results and discussion

We generated steady state photon emission intensity spectra for the nonlinearly treated NV$^\perp$MNP and NV$^\parallel$MNP configurations using the following procedure: The NV centre was initiated in its zero-phonon ground state and evolved using the previously outlined piecewise superoperator method to obtain the steady state NV density matrix $\hat{\rho}_{\rm RF}$. We then obtained the density matrix-dependent steady state Hamiltonian using (8) and (20).

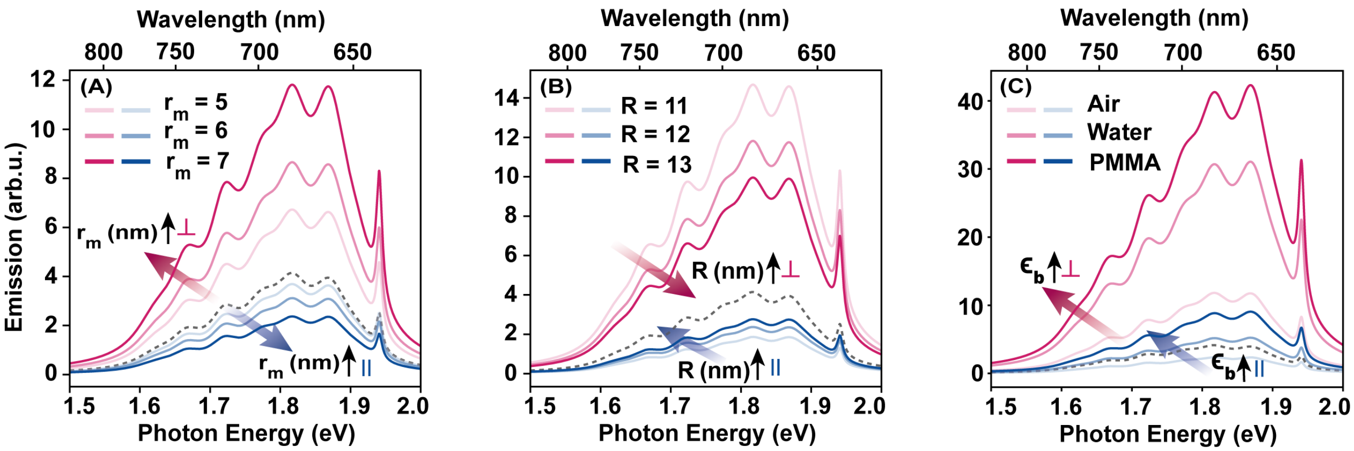

FIG. S1. (A) MNP radius (r_{m}) dependence of total near-field NV emission in the presence of small AuNPs of radii 5, 6 and 7 nm at 12 nm centre separation, in air. (B) MNP centre separation (R) dependence of NV emission in the presence of a 7 nm radius AuNP in air. (C) Submerging medium dependence of NV emission in the presence of a 7 nm radius AuNP at a 12 nm centre separation. The red and blue shaded curves in all three subplots correspond to NVs in $\mathrm{NV}^{\perp}$MNP ($\perp$) and $\mathrm{NV}^{\parallel}$MNP ($\parallel$) configurations, respectively. The dashed reference line corresponds to the emission intensity of the isolated NV centre in air. All curves are normalized by the area of the respective reference curve. Illumination is at the free-space wavelength 532 nm.

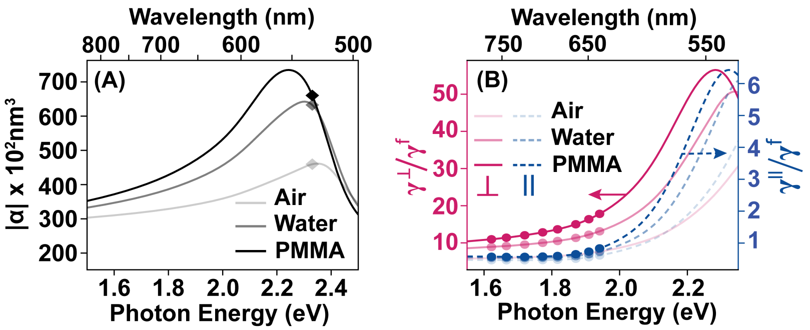

FIG. S2. (A) Absolute polarizabilities $|\alpha|$ for $r_{\mathrm{m}} = 30\,\mathrm{nm}$ AuNP in different media. Diamond markers depict the values at the illumination frequency. (B) Decay rate modification for generic emitters in different media, oriented $\perp$ and $\parallel$ to the surface of the $r_{\mathrm{m}} = 30\,\mathrm{nm}$ AuNP at $R = 38\,\mathrm{nm}$. Circles depict the values at NV emission peaks.

The steady state density matrix-based Hamiltonian and all collapse operators accompanied by their respective rates (arranged in the form $\sqrt{\Gamma_x}\hat{L}_x$) were then input to the *spectrum*() function in QuTiP to obtain the emission correlation spectra for each emission operator $\hat{\sigma}_k$. The total NV emission spectrum in the rotating reference frame was then obtained as the summation of such spectra for all NV emission bands, as outlined in (32). The obtained spectra were normalized by the area of the respective isolated NV emission intensity spectrum and shifted into the laboratory reference frame by adding $\hbar\omega_{\mathrm{d}}$ to the emitted photon energies (in the independent axis).

The same procedure was followed in the absence of MNP induced electric field components, when generating the isolated NV emission spectra in Fig. 1(C) of the main text and the reference curves in all figures.

Sample results generated using the above procedure, for MNP radius (r_{m}), centre separation (R) and submerging medium permittivity (ϵ_{b}) dependence of the NV emission intensity in the presence of small MNPs are depicted in Fig. S1. It is evident that the behavioural trends of NV emission in the presence of small MNPs are qualitatively equivalent to those observed in the presence of large MNPs, presented in the main text.

Absolute polarizabilities of the $r_{\mathrm{m}} = 30\,\mathrm{nm}$ AuNP and decay rate modifications of emitters oriented $\perp$ and $\parallel$ to its surface (at $R = 38\,\mathrm{nm}$) in different media, relevant to Fig. 3 of the main text, can be found in Fig. S2.

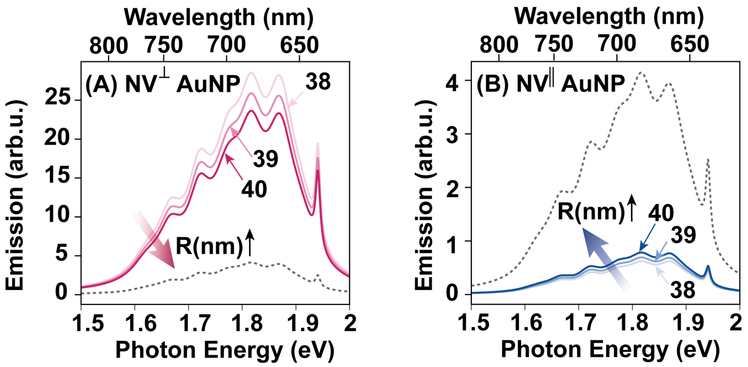

FIG. S3. Variation of the total near-field emission of an NV centre in NV$^\perp$AuNP and NV$^\parallel$AuNP setups in Fig. 3(A) and (B) of the main text for different centre separations (R). The AuNP radius $r_\mathrm{m} = 30\,\mathrm{nm}$ and the background medium is air. The reference (dashed black) curves show the emission of the isolated NV centre in air.

Fig. S3 shows the variation of the total near-field emission of an NV centre in NV$^\perp$AuNP and NV$^\parallel$AuNP setups in Fig. 3(A) and (B) of the main text for different centre separations (R). It is observable from these results that the qualitative impact of reducing the NV-MNP centre separation (R) for a fixed MNP radius is similar to that of increasing the MNP radius at a fixed centre separation. This is because both result in increasing the MNP dipole response field at the NV location. Thus, we observe an increase in NV emission intensity with decreasing R for the NV$^\perp$AuNP setup, and vise-versa for the NV$^\parallel$AuNP setup, in the current parameter region.

[1] M. Fox, *Quantum optics: an introduction*, Vol. 15 (OUP Oxford, 2006).

[2] R. D. Artuso, *The Optical Response of Strongly Coupled Quantum Dot-Metal Nanoparticle Hybrid Systems*, Ph.D. thesis, University of Maryland, College Park, Maryland, United States (2012).

[3] H. Hapuarachchi, S. D. Gunapala, Q. Bao, M. I. Stockman, and M. Premaratne, Physical Review B **98**, 115430 (2018).

[4] H. P. Hapuarachchi, *Analysis of exciton-plasmon nanohybrids*, Ph.D. thesis, Monash University (2019).

[5] J. A. Jones and D. Jaksch, *Quantum information, computation and communication* (Cambridge University Press, 2012).

[6] K. Blum, *Density matrix theory and applications*, Vol. 64 (Springer Science & Business Media, 2012).

[7] J. Steinfeld, *Laser and coherence spectroscopy* (Springer Science & Business Media, 2013) p. 381.

[8] A. Yariv, *Quantum electronics* (Wiley, 1967).

[9] H. Hapuarachchi and J. H. Cole, Physical Review Research **2**, 043092 (2020).

[10] S. A. Maier, *Plasmonics: fundamentals and applications* (Springer Science & Business Media, 2007) Chap. 5.

[11] W. Zhang, A. O. Govorov, and G. W. Bryant, Physical review letters **97**, 146804 (2006).

[12] R. D. Artuso and G. W. Bryant, Nano letters **8**, 2106 (2008).

[13] H. Hapuarachchi, S. D. Gunapala, and M. Premaratne, Journal of Physics: Condensed Matter **31**, 325301 (2019).

[14] S. Raza, S. I. Bozhevolnyi, M. Wubs, and N. A. Mortensen, J. Phys. Condens. Matter **27**, 183204 (2015).

[15] G. C. Des Francs, A. Bouhelier, E. Finot, J.-C. Weeber, A. Dereux, C. Girard, and E. Dujardin, Optics express **16**, 17654 (2008).

[16] G. Colas des Francs, S. Derom, R. Vincent, A. Bouhelier, and A. Dereux, International Journal of Optics **2012** (2012).

[17] G. C. des Francs, J. Barthes, A. Bouhelier, J. C. Weeber, A. Dereux, A. Cuche, and C. Girard, Journal of Optics **18**, 094005 (2016).

[18] R. Carminati, J.-J. Greffet, C. Henkel, and J.-M. Vigoureux, Optics Communications **261**, 368 (2006).

[19] T. Christensen, W. Yan, S. Raza, A.-P. Jauho, N. A. Mortensen, and M. Wubs, Acs Nano **8**, 1745 (2014).

[20] N. A. Mortensen, S. Raza, M. Wubs, T. Søndergaard, and S. I. Bozhevolnyi, Nature communications **5**, 1 (2014).

[21] S. Schietinger, M. Barth, T. Aichele, and O. Benson, Nano letters **9**, 1694 (2009).

[22] M. Pelton, Nature Photonics **9**, 427 (2015).

[23] P. B. Johnson and R.-W. Christy, Physical review B **6**, 4370 (1972).

[24] A. Nisar, H. Hapuarachchi, L. Lermusiaux, J. H. Cole, and A. M. Funston, arXiv preprint arXiv:2109.08537 (2021).

[25] H.-P. Breuer, F. Petruccione, *et al.*, *The theory of open quantum systems* (Oxford University Press, 2002).

[26] H. J. Carmichael, *Statistical methods in quantum optics 1: master equations and Fokker-Planck equations*, Vol. 1 (Springer Science & Business Media, 1999) pp. 35,48,57.

[27] P. Meystre and M. Sargent, *Elements of quantum optics* (Springer Science & Business Media, 2007).

[28] P. D. Nation and J. Johansson, online at http://qutip.org (2011).

[29] A. Hatef, S. M. Sadeghi, and M. R. Singh, Nanotechnology **23**, 065701 (2012).

[30] J. Noye, *Computational techniques for differential equations* (Elsevier, 2000).

[31] S. Barnett, *Matrices: Methods and applications* (Oxford University Press, 1990) Chap. 5.

[32] F. W. Byron and R. W. Fuller, *Mathematics of classical and quantum physics* (Dover Publications, 1992) Chap. 3.

[33] J. R. Johansson, P. D. Nation, and F. Nori, Computer Physics Communications **183**, 1760 (2012).

[34] R. Albrecht, A. Bommer, C. Deutsch, J. Reichel, and C. Becher, Physical review letters **110**, 243602 (2013).

[35] A. Beveratos, R. Brouri, T. Gacoin, J.-P. Poizat, and P. Grangier, Physical Review A **64**, 061802 (2001).

[36] M. W. Doherty, N. B. Manson, P. Delaney, F. Jelezko, J. Wrachtrup, and L. C. Hollenberg, Physics Reports **528**, 1 (2013).

[37] A. Alkauskas, B. B. Buckley, D. D. Awschalom, and C. G. Van de Walle, New Journal of Physics **16**, 073026 (2014).

[38] M. Premaratne and G. P. Agrawal, *Theoretical Foundations of Nanoscale Quantum Devices* (Cambridge University Press, 2021).
